# Biomechanical simulations of crystalline lens oscillations resulting from the changes in the gaze in an accommodated eye

**DOI:** 10.3389/fbioe.2025.1504769

**Published:** 2025-03-05

**Authors:** Ali Dahaghin, Milad Salimibani, Agnieszka Boszczyk, Agnieszka Jóźwik, Jorge Grasa, Joanna Przeździecka-Dołyk, Damian Siedlecki

**Affiliations:** ^1^ Department of Optics and Photonics, Wroclaw University of Science and Technology, Wrocław, Poland; ^2^ Aragón Institute of Engineering Research (I3A), University of Zaragoza, Zaragoza, Spain; ^3^ Centro de Investigación Biomédica en Red en Bioingeniería, Biomateriales y Nanomedicina (CIBER-BBN), Zaragoza, Spain; ^4^ Deanery of Clinical Sciences, University of Edinburgh, Edinburgh, United Kingdom; ^5^ Department and Clinic of Ophthalmology, Wroclaw Medical University, Wroclaw, Poland; ^6^ CREO Research and Development Centre SPEKTRUM Clinical Ophthalmic Center, Wroclaw, Poland

**Keywords:** *in vivo* crystalline lens oscillations, lens inertial motion, lens wobbling, finite element method, Purkinje imaging, ocular biomechanics

## Abstract

**Purpose:**

The goal of the study is to introduce a generic, versatile biomechanical model that aims to reproduce the dynamic wobbling phenomenon.

**Methods:**

A systematic strategy is used, which includes a) capturing the *in vivo* data on a group of healthy volunteers, b) analyzing the changes in Purkinje images over time, and c) performing the combined biomechanical and optical simulations to develop the model that might be useful for understanding the mechanical behavior of the lens during wobbling and its influence on ocular dynamics.

**Results:**

Examples of lens wobbling patterns for six measured eyes were presented, and parameters characterizing the oscillatory motion were determined, including frequency of oscillations, Q-factor, damping factor and time constant. The average values of these parameters are the following: frequency: 20.0 ± 2.4 Hz; Q-factor: 1.86 ± 0.44; damping factor: 0.27 ± 0.06; time constant: 0.11 ± 0.06 s. The data reproduced by means of simulations: frequency: 19.3 Hz; Q-factor: 2.17; damping factor: 0.23; time constant: 0.15 s. This comparison reveals a good agreement between the measured and reconstructed data with the values being within the standard deviation limits.

**Conclusion:**

The developed generic model together with the presented methodology is able to reconstruct the typical crystalline lens wobbling dynamics with a satisfying accuracy. However, the observed intersubject variability highlights the need for personalized biomechanical models. The introduced model may constitute the basis for future individualization of the data, bringing broad perspectives for prospective investigations aimed to explain the biomechanical mechanisms within the eye.

## 1 Introduction

The human eye possesses one of the fastest muscles in the human body, capable of generating 40° rotations in roughly 100 ms (Silva et al., 2020). This rapid movement allows us to quickly shift our gaze towards objects or points of interest in our field of vision. Focusing on objects involves adjusting the focal length of our eyes to make the selected object appear sharp and clear (Wang and Pierscionek, 2019; Cabeza-Gil et al., 2021), a process that becomes less effective with age. This process, called accommodation, is controlled by the contraction and relaxation of the ciliary muscles. When we look at distant object, our lens becomes thinner, the curvatures of its surfaces become smaller; whereas when we look at something near, the lens becomes thicker, and its curvatures increase. These changes in the shape of the lens are accompanied by a change in the tension of the zonular fibers that suspend the lens in place.

The crystalline lens, as a vital component of the eye’s optical system, is also known to display intriguing wobbling movements within the eye. The impact of changing the direction of gaze on the dynamic movement of the crystalline lens is not yet completely understood. This movement is attributed to the lens being suspended by a network of delicate fibers known as zonular fibers. It is described as “inertial oscillatory”, which means that it occurs due to the lens’s inertia, or resistance to changes in motion, and it follows a back-and-forth or oscillatory pattern (Tabernero and Artal, 2014; Pan et al., 2023). These movements may become smaller, and the oscillations may be less apparent in older individuals due to increased tissue stiffness. They also tend to be larger when the eye accommodates (Deubel and Bridgeman, 1995; Nyström et al., 2015). Studies indicate that an optical setup can indirectly measure and study lens wobbling (Tabernero et al., 2016), by utilizing the variations in the locations of the 1st and the 4th Purkinje images, which are reflections of light from the first surface of the cornea (*PI*) and the second surface of the lens (*PIV*). This approach uses Purkinje image eye-tracking techniques to analyze lens wobbling effects indirectly.

Exploring the complexities of crystalline lens wobbling has become a rapidly growing and challenging area of study (Tabernero et al., 2016; Yamagishi et al., 2020; Mardanbegi et al., 2020; Llapashtica et al., 2024). In addition, in modelling research, it is known that the lens wobbling phenomenon can be reproduced either for natural crystalline lens or for artificial implant (Martin et al., 2009; Boszczyk et al., 2023). However, to date, there has not been a sufficient number of research papers that focus on providing an accurate representation of biomechanical simulations based on finite elements (Dahaghin et al., 2024). This gap in the literature can be overcome with the studies presenting a thorough investigation into this critical aspect of eye biomechanics. In this paper, we focus on capturing and analysing Purkinje image trajectories, followed by simulating the mechanical behavior of the lens during wobbling. Subsequently, these findings are used to conduct optical simulations, which aim to simulate a wobbling pattern of the Purkinje images trajectories, caused by the mechanically simulated lens movement. This research provides valuable insights into how the gaze changes affect the dynamic arrangement of the lens, which is manifested as a temporal separation of Purkinje images *PIV* and *PI*.

## 2 Materials and methods

**FIGURE 1:**
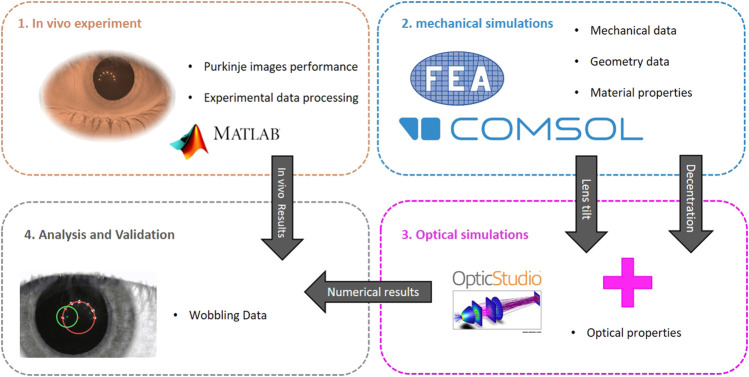
Steps involved in the study and simulation process. The diagram outlines the key stages and their interconnections, providing a visual guide to the overall workflow.

The research study followed a structured approach, as depicted in Figure 1. The first step involved capturing the movement of the 4th and the 1st Purkinje images in response to eye rotation, as well as determining the spatial separation of these images due to lens wobbling.

At this point it is important to note that what one can observe through Purkinje imaging is not the direct motion of the lens itself, but rather the optical effect of this motion—the relative motion of *PIV* and *PI*, which are specular reflections from the posterior surface of the lens and the anterior surface of the cornea, respectively. The information retrieved from Purkinje images reflects only the *PIV-PI* separation, not the exact arrangement of the lens within the eye. Therefore, to derive the physical arrangement of the lens from Purkinje image data, a workflow combining both mechanical and optical simulations need to be applied. This integrated approach allows us to model the lens dynamic behavior and its corresponding optical effects, enabling the extraction of lens arrangement data from the observed Purkinje performance. This methodology, described in detail in our previous work (Boszczyk et al., 2023), bridges the gap between optical observations and mechanical lens dynamics.

Therefore, the next stage of our investigation, we implemented this data workflow, aiming to determine the lens wobbling pattern and its parameters, using finite element simulations under the same conditions as the *in vivo* experimental setup, i.e.,: rotation amplitude, average dimensions, IOP, etc. The output of these mechanical simulations was subsequently utilized as an input for optical simulations in the third stage. The objective of these optical simulations was to illustrate the paths of Purkinje images and to assess the temporal spatial distance between them. Finally, the image separations obtained from the combined mechanical and optical simulations were compared to the *in vivo* data for further analysis and validation.

### 2.1 *In vivo* experiment

#### 2.1.1 Optical setup

**FIGURE 2:**
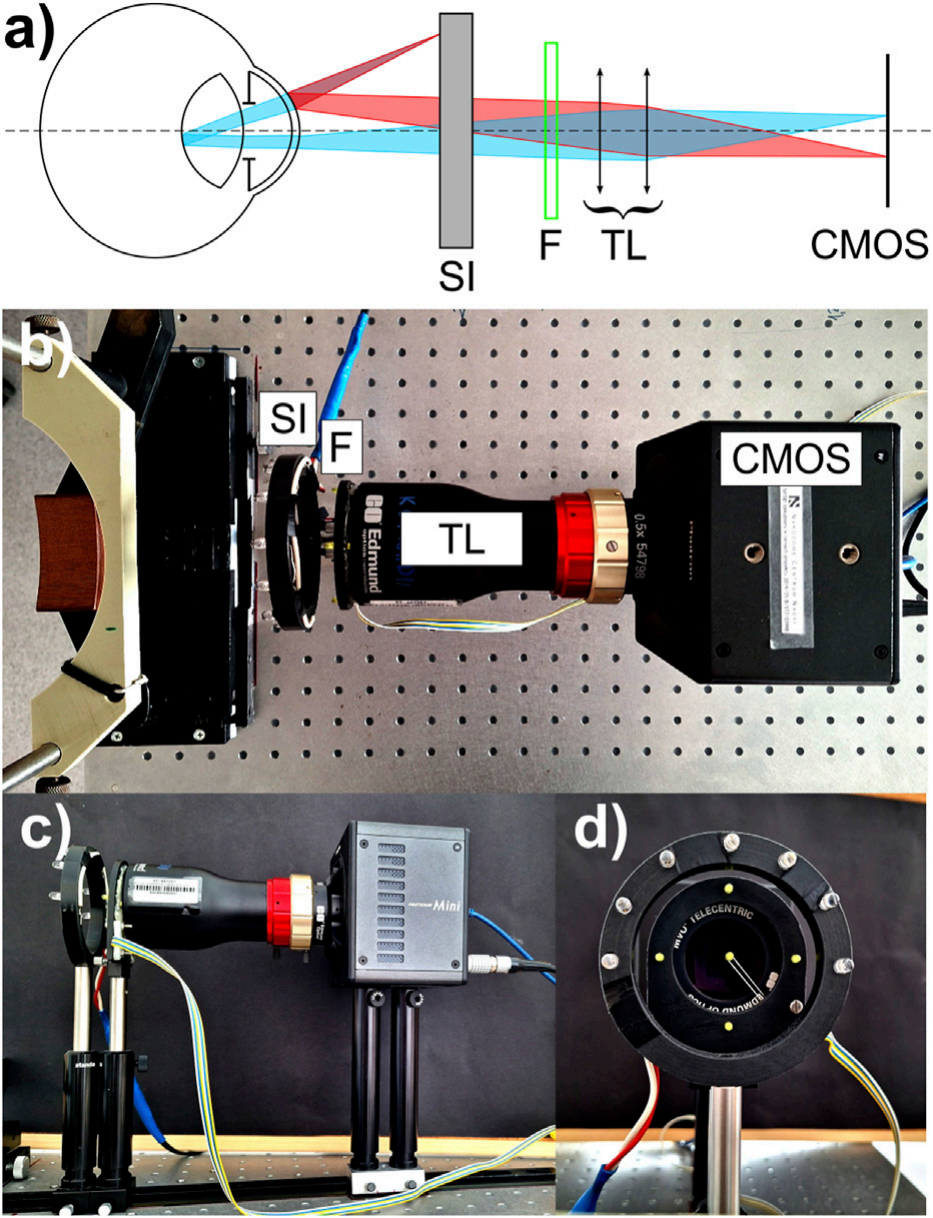
Experimental setup: **(A)** schematic presentation, **(B)** top view, **(C)** side view, **(D)** front view. Explanation of abbreviations: SI, semicircular illuminator; F, fixation target; TL, telecentric lens; CMOS, image sensor (camera).

The *in vivo* data were captured with use of the optical setup (see Figure 2) based on the architecture of a Dynamic Purkinje-meter introduced by Tabernero and Artal (2014). In order to increase both the sampling frequency and the spatial resolution a 1.3-megapixel CMOS image sensor (resolution: 1,280 × 1,024 pixels; pixel size: 10 μm × 10 μm) was incorporated in the compact high-speed camera system (FASTCAM Mini UX50, Photron Limited, Tokyo, Japan). Such a modification enabled us to capture sequences of Purkinje images at the speed of 640 frames per second. The camera was equipped with 0.5× telecentric lens (GoldTL™ line, Edmund Optics), ensuring the constant magnification across the image. The effective size of the pixel on the recorded image was estimated to be 19.5 μm. A semicircular illuminator (SI), consisting of 7 infrared diodes (dominant wavelength of 850 nm), was placed approximately 12 cm in front of the eye. The peripheral fixational yellow diodes were mounted on a plastic plate at an angular distance of 10° from the central diode in order to stimulate the subject to change the gaze (Figure 2B). The fixation target (F) was operated by the software in order to adjust both its brightness and flickering frequency. The flickering frequency between the central and the peripheral diodes was set to 1.25 Hz. This value facilitated multiple saccades between blinks, while also enabling the positions of the Purkinje images to stabilize prior to the subsequent eye movement. The measurements were conducted under conditions of low brightness of fixation target, chosen to ensure that the tracking of the diodes was comfortable for the subject while minimizing the visibility of reflections from the corneal surface. This approach allowed the fixation diode images to remain unobtrusive, thereby not interfering with the readings of the positions of the Purkinje images generated by the infrared diodes.

#### 2.1.2 Human subjects and protocol

In order to obtain the original *in vivo* data for further processing and validation with optical/mechanical simulations outcomes, five subjects (3 males and 2 females), aged 21, 45, 23, 23 and 25 y.o., respectively, took part in the experimental part. Informed consent was obtained from them and the study protocol was in accordance with the guidelines of the Declaration of Helsinki. It was approved by Bioethics Committee of Wrocław Medical University (application No: 2021/0131). The subjects’ eyes were randomly chosen for examination. All the subjects–apart from Subject #5 – were either emmetropic or slightly myopic up to −1.0 D in spherical equivalent. Spherical equivalent of Subject #5 left was equal to −2.5 D. None of the subjects has ever experienced any ocular intervention or treatment. The effective amplitude of accommodation was estimated to be about 8–9 D (5 D for the eldest Subject #3). The location of the near point of accommodation was previously verified to determine whether the person was able to see the target clearly at a distance of 12 cm. Examination was monocular. The subjects were asked to attentively follow the fixation target with the measured eye. The captured video contained a set of up to 4 both temporal-central-nasal and nasal-central-temporal saccades. In order to unify the processing methodology, it was assumed that only the saccades that finished in central position will be selected for further consideration and quantitative analysis.

### 2.2 Simulations

Optomechanical simulation of the crystalline lens refers to a computational modelling approach that combines mechanics (structural properties) and optics (optical propagation and construction of Purkinje images) to simulate the oscillatory dynamics of the lens in response to saccadic eye motion. These simulations refer to computational modelling and analysis of the mechanical movement or vibrations of the crystalline lens in the human eye, taking into account its optical properties.

#### 2.2.1 Mechanical simulations

**FIGURE 3:**
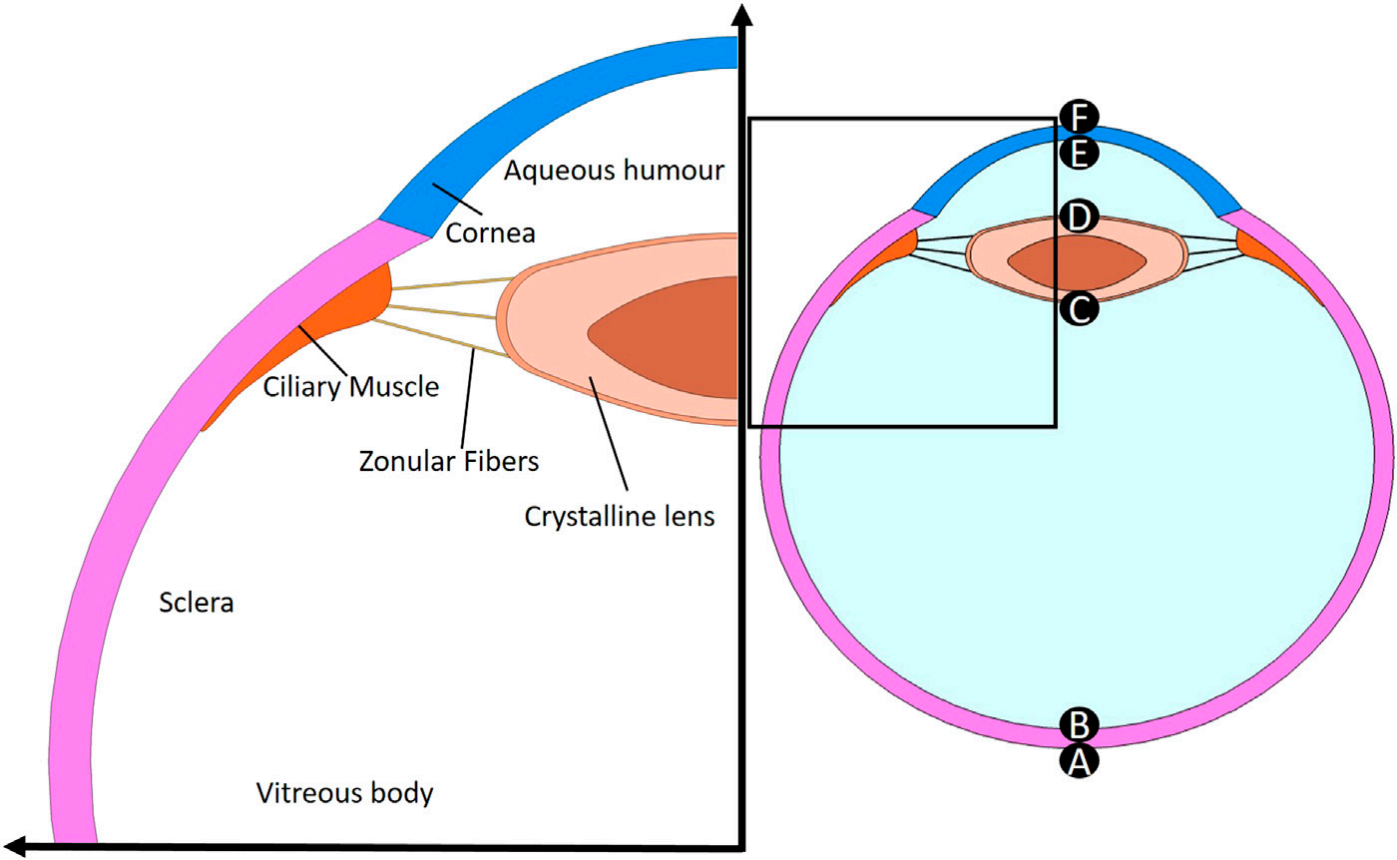
Geometry of the model containing the main and the most influential elements in eye biomechanics: lens, vitreous body, zonular fibers, cornea, sclera, aqueous humour and ciliary muscle. Letters from A to F represent the apical points of the interfaces of the eye.

The mechanical simulation of lens wobbling analyses the intricate dynamics of the lens’ oscillatory motion. Its effective pattern is a complex outcome of the mechanical parameters of the neighbouring ocular structures and their interaction within the eye globe. This simulation aims to accurately replicate the physical behavior of the lens as it wobbles. To conduct these analyses, a 2D numerical model was developed in COMSOL Multiphysics v5.6, using a mechanical design of the eye globe. A 2D model was considered sufficiently precise for this analysis because the Purkinje images generated by lens rotation occur within the same plane, allowing the out-of-plane dimension to be neglected. This model contains the main and most influential elements in eye biomechanics: lens, vitreous body, zonular fibers, cornea, sclera, aqueous humour and ciliary muscle (see Figure 3).

##### 2.2.1.1 Geometry

Accurate representation of the eye’s geometry is a crucial consideration for realistic and reliable Finite Element simulations. In this study, all the geometry data was adopted from an accommodated and age-dependent optical eye model based on *in vivo* measurements presented by Zapata-Díaz et al. (2019).

The most commonly used optical surface in the eye is a conic surface. For surfaces, such as those found in lens and cornea and when working with a cylindrical coordinate system, the *z*-coordinate is given by an equation that depends on the specific conic shape. The *z*-coordinate of the standard surface depends on the type of surface is used and is determined by Equation (1):

$$z=\frac{{cr}^{2}}{1+\sqrt{1-\left(1+k\right)c^{2}r^{2}}}$$ (1)

where *c* represents the curvature, *r* stands for the radial coordinate, and *k* is the conic constant.

Table 1 shows the calculated model parameters for 20 years old eye, with an accommodation state of 2.5 diopters. The total distance between the posterior surface of the eyeball and the anterior cornea is typically referred to as the axial length of the eye, which here is 24 mm.

**TABLE 1:** Summary of geometrical parameters extracted from Zapata-Díaz et al. (2019) studies for 20 years old eye with an accommodation state of 2.5 dioptries.

Modelled structure	Radius [mm]	Thickness^a^[mm]	Refractive index	Conic constant
Anterior corneal surface	7.87	0.574	1.376	−0.18
Posterior corneal surface	6.40	2.996	1.336	−0.12
Anterior lens surface	9.825	3.522	1.4332	−7.05
Posterior lens surface	−5.649	16.908	1.336	−4.6
Retina	−12.9	n.a.	n.a.	0.193

^a^ Central thickness to the next surface.

The values marked with blue colour were subject to change with accommodation.

**FIGURE 4:**
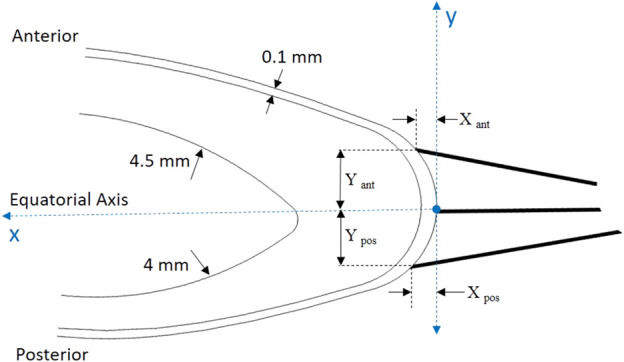
Position of the connection points where zonular fibers are attached in the capsule.

The lens is suspended in place by thin zonular fibers that attach to the lens capsule and extend to the ciliary body. Due to their different locations three zones are distinguished: anterior, equatorial, and posterior (Table 2). Together, these fibers create a support system that keeps the lens in place while allowing it to change shape as needed for clear vision at different distances. This strategy follows the approach of Lanchares et al. (2012), using the three connection points to attach the idealized zonular fibers and replicating their methodology for modeling fiber attachment to the lens and ciliary body, although Lanchares do not explicitly represent the fibers themselves but rather the direction of the forces they exert. Figure 4 presents the arrangement of the fibres on the lens capsule, with the dimensions shown in Table 2 used to position them according to the reference system located at the lens equator.

**TABLE 2:** Zonular fibers arrangement data. Connection points and thickness of the band.

Zonular fibers	X [mm]	Y [mm]	Thickness [mm]
Anterior	0.34	0.67	0.40
Equatorial	0.00	0.00	0.50
Posterior	0.26	0.74	0.50

The minimal thickness of the capsule is 0.1 mm (Dai et al., 2017); other dimensions from the literature was employed for forming the lens’ nucleus (Cabeza-Gil et al., 2021).

##### 2.2.1.2 Material properties

The choice of material properties depends on the type of analysis being conducted, which varies based on the specific focus of the research, such as the deformation of particular tissues or, in our case, the dynamic response of the lens to the rotational motion of the eye.

To replicate the physiological conditions, vitreous body and aqueous humour are both modeled as viscous Newtonian incompressible fluids with constant pressure.This means that their behavior follows Newton’s law of viscosity, where the shear stress is directly proportional to the rate of deformation (velocity gradient). Dynamic viscosity, which represents the resistance of fluids to flow under an applied shear stress, and density are, respectively, 0.00074 Pa·s and 1,000 kg/m^3^ (Singh et al., 2017), which are assumed to be constant throughout the simulation.

In this study, other tissues are treated as linear elastic, isotropic, and homogeneous. This means that the materials were assumed to respond to mechanical stress linearly and have the same mechanical properties in all directions. Although their true behavior deviates from this idealization, this assumption remains valid for this first approach, as the focus of the study is on the lens movement, and the tissues will not experience large deformations. This approach allows us to simplify the model and focus on specific mechanical aspects relevant to the study. Different material properties for a normal eye are considered, to provide a comprehensive understanding of the mechanical properties. Table 3 presents the values of Young’s modulus, Poisson’s ratio, and density for the different eye components used in FE simulations.

**TABLE 3:** Mechanical properties assigned to ocular tissues (Cabeza-Gil et al., 2021; Bocskai and Bojtár, 2013; Issarti et al., 2021; Osmers et al., 2021).

Modelled tissue		Young’s modulus [kPa]	Poisson’s ratio [-]	Density [kg/m^3^]
Ciliary Muscle		350	0.47	1,225
Crystalline lens	Capsule	1,000	0.49	1,078
	Cortex	3	0.49	1,078
	Nucleus	0.3	0.49	1,078
Sclera		3,000	0.47	1,400
Cornea		400	0.42	1,400
Zonular Fibers		1,500	0.49	1,000

##### 2.2.1.3 Boundary conditions

The sclera is the white outer layer of the eyeball. It is a tough and fibrous tissue that provides the eyeball with structural support. During eye movement, it was assumed that the shape and size of the sclera remained constant and did not undergo any deformation; additionally, its center, acting as the pivot point for the rotation of the eyeball, remains fixed and does not undergo any linear movement. It underwent a 10 deg rotation (angular movement), and the angular velocity was assumed to follow the 10 deg range gaze profile reported by Collewijn et al. (1988) with the peak velocity of 300 deg/s (c.a. 5.67 rad/s). Throughout this movement, mechanical displacement data were recorded for the apical points noted as A to F in Figure 3. Under the assumption of negligible deformation of the cornea and lens due to rotation, the apical points A-F provide sufficient information regarding the arrangement (tilt and decentration) of the lens within the eye globe. This allows for the optical simulation of the location of Purkinje images in accordance with Equation 2.

The eye contains a clear fluid that helps to maintain the eye globe shape by intraocular pressure (IOP). IOP plays a crucial role, therefore any changes in it can have an impact on the ocular structure and may be associated with certain diseases. To simulate the normal conditions in the described model, an initial pressure of 15 mmHg was set for the fluid part (Sarmiento et al., 2023). This value is within the typical range in a healthy eye. This pressure was applied to the inner surface of the sclera, lens, and ciliary body.

##### 2.2.1.4 Mesh

Triangular elements are particularly useful for analyzing irregular or complex geometries, so to discretize both the solid and fluid domains, those elements were employed. A built-in quality assessment was used to evaluate the element quality, which is based on the equiangular skew. This assessment assigns a rating between 0 and 1, where a quality above 0.5 is considered acceptable (Etminan et al., 2023). This model contains a total of 44,522 elements and has an average element quality of 0.82. This optimal mesh size was selected after performing a sensitivity analysis of the model. It was found that reducing the global mesh size parameter in the program did not significantly affect the outcome.

#### 2.2.2 Optical simulations

To verify the experiment with the mechanical simulation results, an optical model of the eye including an illumination and a Purkinje reflection imaging system was created using Zemax OpticStudio (Zemax, 2024) software. The non-sequential software mode was used for this purpose. Detailed information about the designed system can be found in our previous paper (Boszczyk et al., 2023), with the modification that the parameters of the accommodated eye (Table 1) were used for the model, not the relaxed one. The output of the mechanical simulations (changes of *x* and *y* coordinates of each eye surface during the time) was used as an input data for optical simulations. The result of optical simulations are images showing the first and fourth Purkinje reflections (*PI* and *PIV*), corresponding to individual configurations of the eye surface positions obtained in mechanical simulations.The created optical model allows for determining a simple linear relationship between different alignments of the crystalline lens and the difference in the positions of *PIV-PI* images, when the eye (cornea) is positioned in front of the imaging system. For this purpose, images showing the corresponding Purkinje reflections were simulated for various lens locations and orientations. Then, the positions of these images were established through a systematic process. Initially, the centroids of the *PI* and *PIV* images corresponding to each diode were identified. Subsequently, two circles were fitted to these centroids—one for the *PI* reflections and another for the *PIV* reflections. The position of each Purkinje image was then defined as the center of the fitted circle.

## 3 Results

The relationship between the distance of Purkinje images (*PIV-PI*) and the position of the lens (derived through a linear fit of data estimated by means of optical simulations with R^2^ = 0.999), took a form:

PIV−PI=0.04147 tilt−1.059 dec−0.003 (2)

where *PIV-PI* is expressed in mm, *dec*–a decentration of the anterior surface of the lens from corneal optical axis is expressed in mm, and lens *tilt* relative to an axis perpendicular to the corneal axis is expressed in degrees. The standard deviations for the determined coefficients were 0.00029 for *tilt*, 0.003 for *dec*, and 0.002 for the intercept, respectively. The above equation allowed for simplification and acceleration of the comparative analysis for various mechanical simulations with the experiment, without the need to perform optical simulations and detect the positions of Purkinje reflections for each mechanically simulated lens wobbling patterns.

**FIGURE 5:**
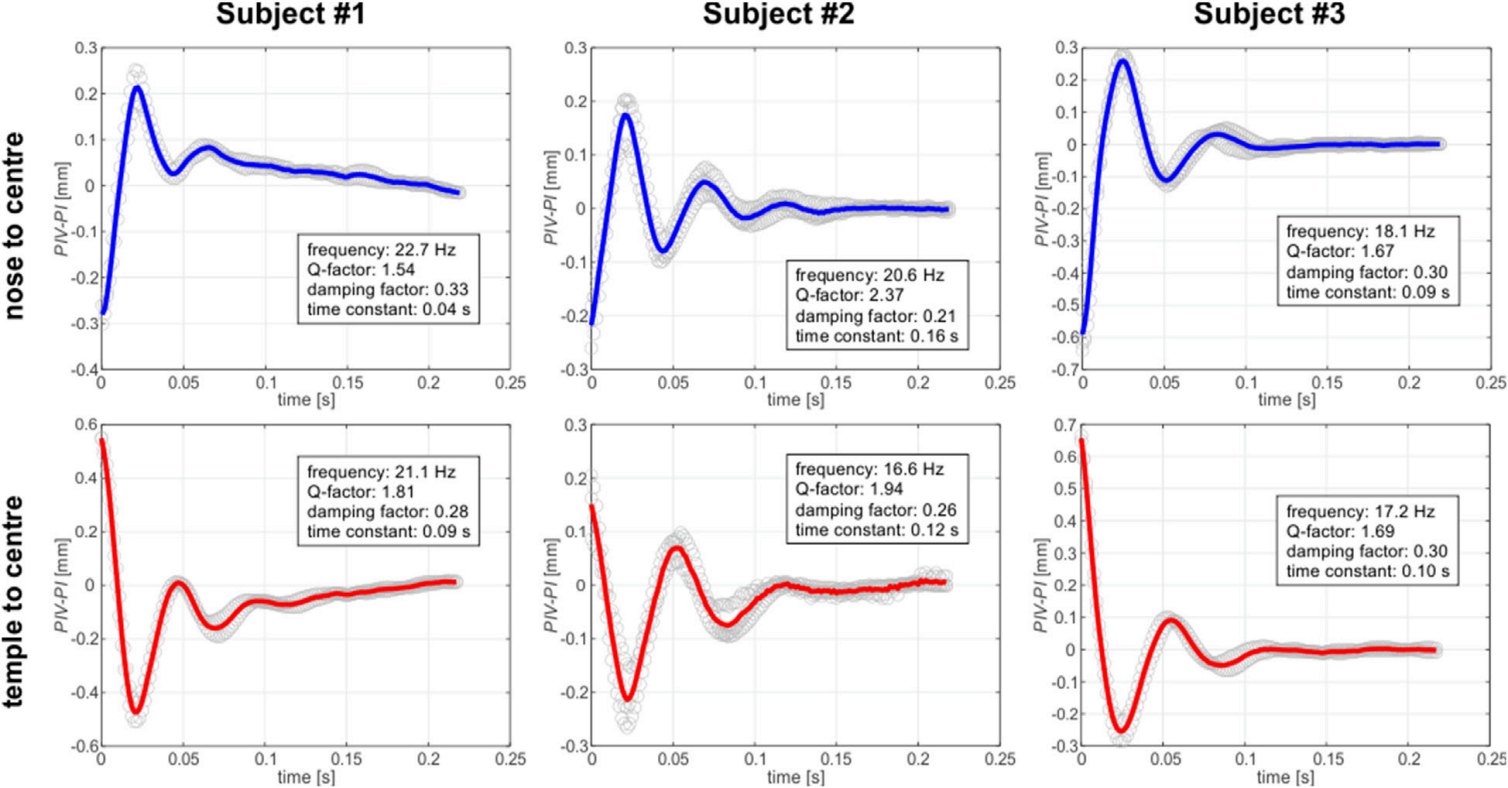
Wobbling patterns for captured for right eye (ER) of three different subjects, depending on the saccade direction. Each plot displays the *PIV-PI* separation as a function of time. Gray open circles correspond to the experimental data. Each single experimental dataset was shifted up or down so that the average level of the last 30 datapoints was equal to zero. Solid lines correspond to mean pattern of the experimental data: blue one for the nose-to-center direction of the gaze and the red one for the temple-to-center direction.

**FIGURE 6:**
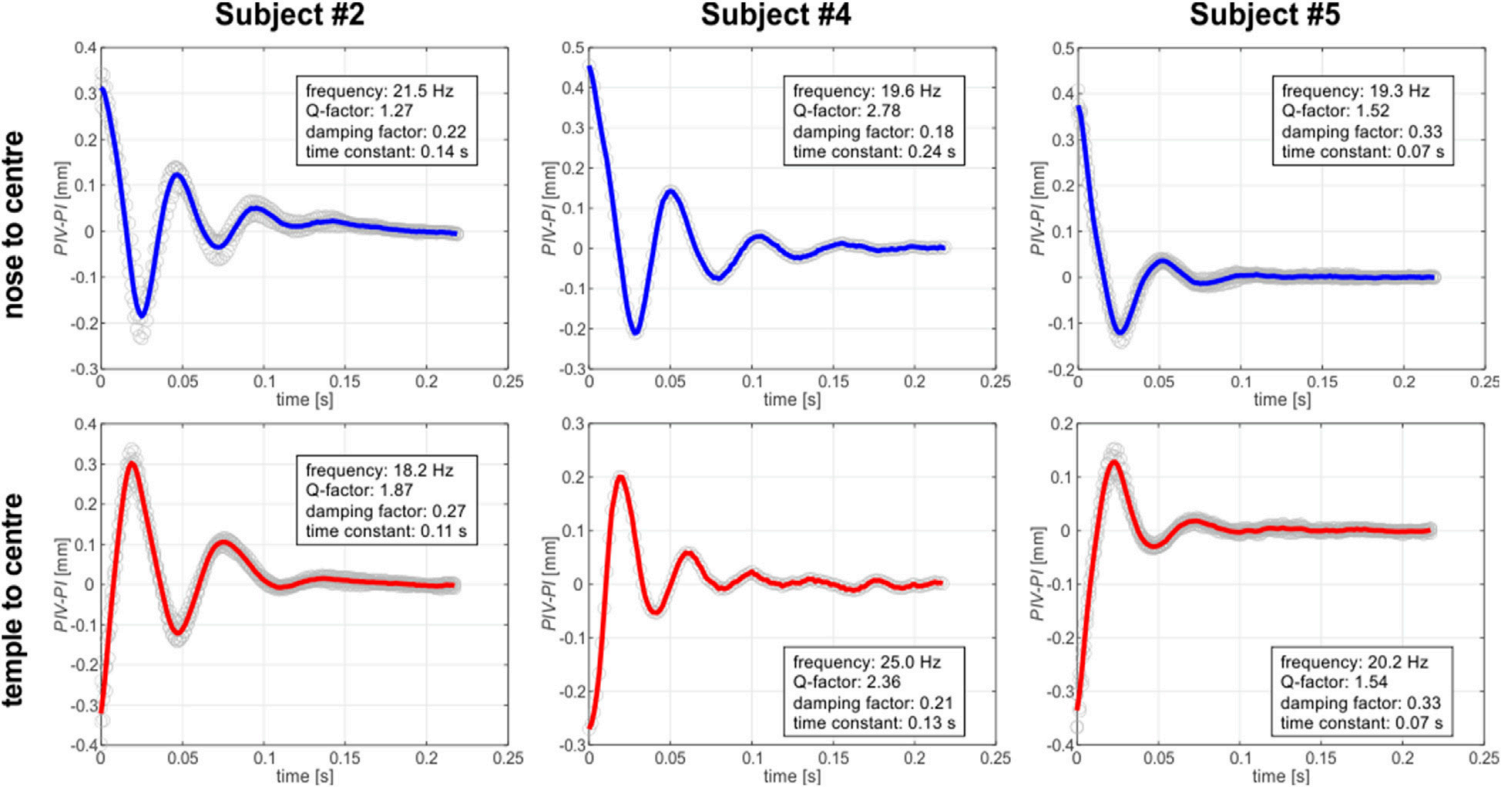
Wobbling patterns for captured for left eye (EL) of three different subjects, depending on the saccade direction. Each plot displays the *PIV-PI* separation as a function of time. Gray open circles correspond to the experimental data. Each single experimental dataset was shifted up or down so that the average level of the last 30 datapoints was equal to zero. Solid lines correspond to mean pattern of the experimental data: blue one for the nose-to-center direction of the gaze and the red one for the temple-to-center-direction.

For the *in vivo* experiment, the whole video recording captured for each of the subject was divided into two sets of sequences: one manifesting the rotation of the eye from the nose to the center and one presenting the motion from the temple to the center. Each set contained up to four sequences. Then, each sequence was then processed in order to retrieve the relative displacement of *PIV-PI* position that is associated with the arrangement of the crystalline lens within the eye globe. As it can be seen on an exemplary sequence video (Supplementary Material S1) the relative distance between *PIV* and *PI* Purkinje images varies with time (Figures 5, 6) so as the crystalline lens undergoes some fine, evanescent motion due to ocular inertia. It should be noted that on each of these plots the time = 0 s was set to the moment, when the maximum distance (maximum wobbling) betwee*n PIV* and *PI* appeared. It can be seen that over time, the wobbling in these graphs is settling down. The outcomes imply that the eye rotations, both from the nose to the center and from the temple to the center, manifest consistent and stable behavior across the tested sequences.

As Tabernero and Artal (2014) pertinently noted, the crystalline lens wobbling motion can be successfully described by a harmonic oscillator characteristics, therefore we decided to use this description in analysis of the results (see the text boxes in Figures 5, 6). The focus of the analysis was on comprehensively examining key parameters including frequency, Q-factor, damping factor, and time constant. The variability of the parameters revealed distinct features for each individual subject and gaze direction, highlighting the importance of further investigation of the factors that influence energy dissipation (Behling et al., 2021) and temporal characteristics during these rotational movements.

As for frequency stability, rotations in both directions displayed roughly stable average frequencies, suggesting consistent oscillatory behavior. Q-factor variations highlight differences in energy dissipation during rotations, emphasizing the intricate nature of the eye’s biomechanics. Intersubject variations of damping factors showed ability of the individual’s eye to dampen oscillations, contributing to overall stability.

The averaged frequencies estimated for the examined group of subjects have comparatively small standard deviations (equal to 12%), suggesting relative stability in the lens oscillatory behavior across examined individuals (Table 4). The mean Q-factors show a moderate level of variability, suggesting differences in energy dissipation between the subjects. Damping factors exhibit the relative standard deviation of about 20%, implying intersubject variations in ability to dampen lens oscillations. However, when one takes into account the damping factors estimated in both directions for each individual, a relative consistency can be observed. Also, a similar observation can be conducted in case of time constants, which manifest rather large standard deviations for the group as a whole, but for each particular subject its values remain relatively constant for both gaze directions. This may suggest a consistent temporal response in the eye dynamics for each individual during rotations.

**TABLE 4:** Averaged parameters of the wobbling trajectories for the whole group of subjects.

	Mean	Std dev	Range (min÷max)
Frequency [Hz]	20.0	2.4	(16.6÷25.0)
Q-factor	1.86	0.44	(1.27÷2.78)
Damping factor	0.27	0.06	(0.18÷0.33)
Time constant [s]	0.11	0.06	(0.04÷0.24)

**FIGURE 7:**
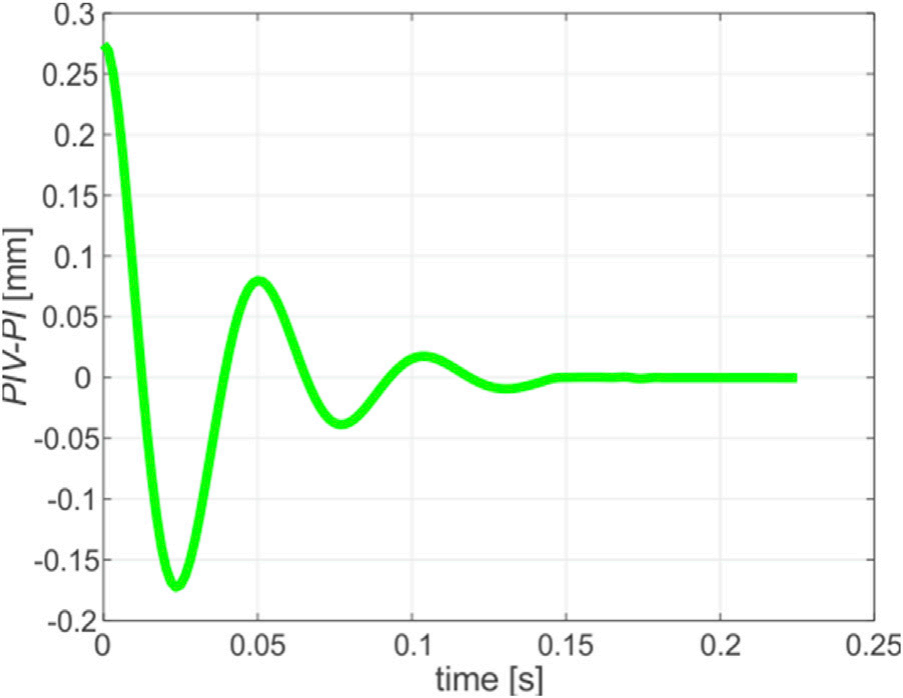
Wobbling trajectory obtained by means of combined FEM and opticals simulations.

The previous sections presented a structured summary of the major results from the experimental analysis of lens displacement data over a specific time period, suggesting potential avenues for further discussion. The combined opto-mechanical simulations that utilize the biomechanical model, display a notable resemblance to the experimental data, as illustrated in Figure 7. The simulated data was processed similarily to the graphs presented in Figures 5, 6: the time stamp equal to zero was set to the moment, when the maximum separation between *PIV* and *PI*, associated with the maximum wobbling amplitude, appeared. By analyzing the temporal evolution of ocular dynamics through lens displacement, a comprehensive understanding is achieved. At the time = 0, a significant positive displacement is observed, which indicates transient relocation of the lens with respect to the cornea regardless of immediate biomechanical interactions within the eye. In particular, periodic variations in lens displacement indicate continuous diminishing motion of the lens.

To assess the effectivity of simulation, a comparison of the wobbling parameters calculated from simulation to the average ones estimated from experiment was performed. Furthermore, to provide clarity, the relative discrepancies were calculated. All these data are presented in Table 5.

**TABLE 5:** Comparison of the averaged wobbling motion parameters estimated from *in vivo* experiment and obtained by means of biomechanical simulations.

	Frequency [Hz]	Q-factor	Damping factor	Time constant [s]
*In vivo* (average data)	20	1.86	0.27	0.11
Simulations	19.3	2.17	0.23	0.15
Relative discrepancy	3.5%	16%	15%	36%

## 4 Discussion

The exploration of ocular dynamics during horizontal eye rotations has revealed intriguing insights into the biomechanics of the human eye. The discussion encompasses the observed patterns in key parameters, their implications, and avenues for future research. Additionally, the documented changes in lens displacement are likely to have a pivotal function in visual accommodation and the eye’s aptitude to concentrate on objects positioned at different distances (Seely and Macklem, 2004). When the simulation data are examined along with experimental observations, valuable insights can be obtained regarding the precision of the ocular rotation model. Acknowledging the use of homogeneous, linear elastic constitutive laws in modeling solid ocular tissues is crucial, as this represents a conscious simplification intended to balance computational efficiency with the essential biomechanical behavior of the eye under the study’s specific conditions. While this methodology simplifies the model and aligns with the study’s aims, it does not adequately address the anisotropic or viscoelastic properties of certain tissues, including the cornea and sclera. Moreover, the sensitivity analysis (Salimibani et al., 2024) emphasizes the need for refining biomechanical models to boost their accuracy and applicability, thus paving the way for future initiatives to tailor simulations for more accurate ocular diagnostics.

The oscillatory behavior of the eye is distinguished by variations in both amplitude and frequency, underscoring the intricate and complex interaction between the cornea and lens. As time progresses, the lens exhibits adaptive resilience, allowing it to rebound from the initial negative displacement and establish a dynamic equilibrium. This resilience observed in the eye signifies its extraordinary capacity to uphold its structural integrity and functionality even in the face of external disturbances arising from rotation. The experimental runs were effectively replicated by the simulation model, thereby showcasing its ability to accurately capture the oscillatory behavior of the eye. This alignment significantly enhances the usefulness of the model in predicting the dynamics of the eye during rotational motions. The agreement in Q-factor and damping factors between the simulation and experimental data further validates the model’s precision in representing the mechanical response of the eye. This consistency greatly contributes to the reliability of the simulation in simulating real-world conditions.

The influence of mechanical parameters on the wobbling and overshooting behavior has been previously examined on an example of *ex-vivo* porcine eye model (Salimibani et al., 2024). The findings of this study indicate that while the effects of structures not in close proximity to the crystalline lens are minimal, changes in the mechanical characteristics of the zonular fibers play a crucial role. These results are likely applicable to the *in vivo* human eye model. However, the presence of additional anatomical features, such as the capsular bag and intricate lens components, adds a layer of complexity. This underscores the necessity for a thorough sensitivity analysis in future research to improve model customization.

The rationale for utilizing a 2D numerical model is based on its proficiency in accurately depicting the primary horizontal and vertical saccadic motions observed in controlled experimental environments. Nonetheless, we have to clearly acknowledge the assumption of planar eye rotation as a limitation, realizing that real-world saccades may present minor deviations from perfect planarity, which could result in discrepancies between the predictions of the model and the experimental findings.

The strong agreement between the simulated frequencies and the mean frequencies measured experimentally for a group of healthy volunteers demonstrates high reliability of the simulation in accurate reproducing of the oscillatory patterns observed in the eye, as presented in Table 5. Although the simulation quite closely approximates the frequencies, some slight deviations are observed. Taking into account the inherent variability in biological systems (Seely and Macklem, 2004), the discrepancies are considered to be within a reasonable range. The agreement in Q-factor and damping factors between the simulation and experimental runs, as shown in Table 5, is particularly noteworthy. The comparable levels of energy dissipation and stability further support the accuracy of the simulation in capturing the mechanical response of the eye.

A system characterized by consistent damping factors demonstrates its robustness in effectively attenuating oscillations. This resilience greatly contributes to the functional stability of the eye during rotations, accentuating its remarkable adaptability and ability to maintain equilibrium. The consistency in time durations suggests a predictable temporal response in the dynamics of the eye. The deviations observed in individual sequences prompt the need for personalized approaches in ocular biomechanics.

In each sequence, the Q-factors varied, indicating different dissipations of energy. We can gain a deeper understanding of rotational energy dynamics by investigating factors that influence Q-factor variations. As a result, the damping factors exhibited consistency, suggesting that the eye dampens oscillations on a stable basis. Furthermore, the results of this study indicate that the damping factor still exists, albeit with a smoother and altered function. This further supports the idea of the viscoelastic behavior of eye zonulas, as the presence of the damping factor in the results strengthens this concept (Dahaghin et al., 2023).

The presence of consistent damping factors indicates the existence of a robust system that effectively dissipates energy, thereby contributing to the functional stability of the eye during rotations. This discovery holds immense significance in understanding the eye’s ability to withstand perturbations. The uniformity observed in the time constants further suggests a predictable temporal response exhibited by the eye’s dynamics during rotations. When these time constants are understood, it becomes possible to improve predictive models for ocular biomechanics and potentially apply this knowledge in clinical scenarios.

Frequencies showed consistent oscillatory behavior, indicating stable oscillations. However, individual sequences showed variability. On the basis of the stable mean frequencies, it appears that the eye oscillates during rotation fundamentally. As a result of observed variability, personalized approaches in ocular biomechanics are necessary (Navarro et al., 2006). Consequently, comparing the *in vivo* cases with the simulation results shows differences in errors, which means that these parameters can be subjective and vary from one individual to the other. From a certain point of view, everybody has eyes with different geometrical and mechanical properties, as well as different experimental environments that can cause different results compared to simulations.

Personalizing biomechanical models to match individual characteristics can potentially improve the precision of diagnostic assessments and provide valuable guidance for interventions, especially in situations where the biomechanics of the eye are pivotal. The discussion highlights the complex dynamics of ocular biomechanics during specific rotations. The eye’s response is characterized by stable mean frequencies, varying Q-factors, consistent damping factors, and uniform time constants, collectively contributing to the intricate nature of its behavior. The 2D model illustrated in this study effectively demonstrates the *in vivo* outcomes, however, it fails to capture the complexities of the eye behavior in three-dimensional space. This simplification overlooks crucial elements such as torsional eye movements and depth perception, resulting in less accurate predictions. Additionally, the model lacks a detailed representation of the viscoelastic and hyperelastic properties of the tissues, including the cornea, sclera, and ocular muscles. This deficiency impedes the accurate simulation of parameters like frequency, Q-factor, damping factor, and time constant, limiting the relevance of the model to real-world scenarios. Furthermore, while the study assumes the sclera is non-deformable, this may influence the observed lens behavior following eye rotation. Future research should explore the potential impact of scleral deformability on our findings to provide a more comprehensive understanding of ocular dynamics. Moreover, to achieve a more precise and comprehensive analysis, transitioning to a 3D model that incorporates these biomechanical properties is necessary.

## 5 Conclusion

The research highlights the complex interplay between simulation data and experimental observations, with a particular focus on the ongoing refinement of the models. To ensure the accurate simulation of observed dynamics and the validation of experimental outcomes across diverse conditions, advanced biomechanical models have been developed. The proposed methodology has proven its capability to reproduce complex oscillation phenomena using a sophisticated model. A comprehensive understanding of the temporal dynamics involved in lens displacement plays a crucial role in advancing vision science and refining ocular biomechanical models.

As a methodical approach, integrating the biomechanics of the ocular system, has been implemented to address inconsistencies in Q-factors, damping factors, and time constants. Through the replication of experimental outcomes via simulation, we are empowered to explore ocular dynamics in scenarios that may present challenges when attempting to replicate them in a laboratory setting.

Despite the positive results, it is essential to acknowledge specific limitations. A simplification of the model to two dimensions, variations in experimental conditions, subject-specific factors (such as the ability to immediately fixate and maintain gaze on the target, the ability for accommodation, and the extent of accommodative effort), and the potential influence of measurement techniques may introduce uncertainties. Future investigations could prioritize enhancing the simulation model to incorporate additional biomechanical complexities. The customization of biomechanical models to suit individual characteristics holds the potential to greatly benefit personalized medicine and clinical interventions, leading to significant implications in the field.

## Data Availability

The original contributions presented in the study are included in the article/Supplementary Material, further inquiries can be directed to the corresponding author.

## Supplementary material

SUPPLEMENTARY VIDEO S1 Purkinje video sequences (nose to center).

SUPPLEMENTARY VIDEO S2 Purkinje video sequences (temple to center).

SUPPLEMENTARY VIDEO S3 Crystalline Lens Oscillations animation.

## References

[B1] Behling A. V. Giandolini M. von Tscharner V. Nigg B. M. (2021). Soft-tissue vibration and damping response to footwear changes across a wide range of anthropometrics in running. PLoS One 16, e0256296. 10.1371/journal.pone.0256296 34403445 PMC8370632

[B2] Bocskai Z. Bojtár J. (2013). Biomechanical modelling of the accommodation problem of human eye. Period. Polytech. Civ. Eng. 57, 3–9. 10.3311/PPci.2136

[B3] Boszczyk A. Dębowy F. Jóźwik A. Dahaghin A. Siedlecki D. (2023). Complexity of crystalline lens wobbling investigated by means of combined mechanical and optical simulations. Biomed. Opt. Express 14, 2465–2477. 10.1364/BOE.488176 37342700 PMC10278604

[B4] Cabeza‐Gil I. Grasa J. Calvo B. (2021). A validated finite element model to reproduce Helmholtz’s theory of accommodation: a powerful tool to investigate presbyopia. Ophthalmic Physiol. Opt. 41, 1241–1253. 10.1111/opo.12876 34463367

[B5] Collewijn H. Erkelens C. J. Steinman R. M. (1988). Binocular co-ordination of human horizontal saccadic eye movements. J. Physiol. 404, 157–182. 10.1113/jphysiol.1988.sp017284 3253429 PMC1190820

[B6] Dahaghin A. Salimibani M. Boszczyk A. Jozwik A. Siedlecki D. (2023). “Investigation of the crystalline lens inertial overshooting: *ex vivo* and simulation results,” in Abstract retrieved from Proceedings of the COMSOL Conference 2023 Munich, Munich, Germany, 25–27 October, 2023.

[B7] Dahaghin A. Salimibani M. Boszczyk A. Jóźwik A. Skrok M. Grasa J. (2024). Investigation of crystalline lens overshooting: *ex vivo* experiment and optomechanical simulation results. Front. Bioeng. Biotechnol. 12, 1348774. 10.3389/fbioe.2024.1348774 38655391 PMC11035874

[B8] Dai P. Zhao Y. Sheng H. Li L. Wu J. Han H. (2017). Simulating the effects of elevated intraocular pressure on ocular structures using a global finite element model of the human eye. J. Mech. Med. Biol. 17, 1750038. 10.1142/S0219519417500385

[B9] Deubel H. Bridgeman B. (1995). Fourth Purkinje image signals reveal eye-lens deviations and retinal image distortions during saccades. Vis. Res. 35, 529–538. 10.1016/0042-6989(94)00146-D 7900293

[B10] Etminan A. Salimibani M. Dahaghin A. Haghpanahi M. Maleki A. (2023). FEM thermal assessment of a 3-D irregular tumor with capillaries in magnetic nanoparticle hyperthermia via dissimilar injection points. Comput. Biol. Med. 157, 106771. 10.1016/j.compbiomed.2023.106771 36924733

[B11] Issarti I. Koppen C. Rozema J. J. (2021). Influence of the eye globe design on biomechanical analysis. Comput. Biol. Med. 135, 104612. 10.1016/j.compbiomed.2021.104612 34261005

[B12] Lanchares E. Navarro R. Calvo B. (2012). Hyperelastic modelling of the crystalline lens: accommodation and presbyopia. J. Optom. 5, 110–120. 10.1016/j.optom.2012.05.006

[B13] Llapashtica E. Sun T. Grattan K. T. V. Barbur J. L. (2024). Effects of post-saccadic oscillations on visual processing times. PLoS One 19, e0302459. 10.1371/journal.pone.0302459 38809939 PMC11135737

[B14] Mardanbegi D. Wilcockson T. D. W. Killick R. Xia B. Gellersen H. Sawyer P. (2020). A comparison of post-saccadic oscillations in European-born and China-born British university undergraduates. PLoS One 15, e0229177. 10.1371/journal.pone.0229177 32097447 PMC7041864

[B15] Martin H. Bahlke U. Guthoff R. Rheinschmitt L. Schmitz K. P. (2009). “Determination of inertia forces at an intraocular lens implant during saccades,” in World Congress on Medical Physics and Biomedical Engineering, IFMBE Proceedings, Munich, Germany, September 7-12, 2009. Editors Dössel O. Schlegel W. C. (Berlin, Heidelberg: Springer), 100–103. 10.1007/978-3-642-03891-4_27 25/11

[B16] Navarro R. González L. Hernández-Matamoros J. L. (2006). On the prediction of optical aberrations by personalized eye models. Optom. Vis. Sci. 83, 371–381. 10.1097/01.opx.0000221399.50864.c7 16772895

[B17] Nyström M. Andersson R. Magnusson M. Pansell T. Hooge I. (2015). The influence of crystalline lens accommodation on post-saccadic oscillations in pupil-based eye trackers. Vis. Res. 107, 1–14. 10.1016/j.visres.2014.10.037 25481633

[B18] Osmers J. Kaiser N. Sorg M. Fischer A. (2021). Adaptive finite element eye model for the compensation of biometric influences on acoustic tonometry. Comput. Methods Programs Biomed. 200, 105930. 10.1016/j.cmpb.2021.105930 33486338

[B19] Pan Y. Liu Z. Zhang H. (2023). Research progress of lens zonules. Adv. Ophthalmol. Pract. Res. 3, 80–85. 10.1016/j.aopr.2023.02.002 37846380 PMC10577871

[B20] Salimibani M. Dahaghin A. Boszczyk A. Grasa J. Siedlecki D. (2024). Assessment of material properties in key components of the porcine crystalline lens during overshooting. Acta Bioeng. Biomech. 26. 10.37190/ABB-02463-2024-03 40100999

[B21] Sarmiento D. M. M. Montaño Ó. L. R. Castiblanco J. D. A. Rodríguez C. J. C. (2023). The impact of horizontal eye movements versus intraocular pressure on optic nerve head biomechanics: a tridimensional finite element analysis study. Heliyon 9, e13634. 10.1016/j.heliyon.2023.e13634 36865452 PMC9970910

[B22] Seely A. J. Macklem P. T. (2004). Complex systems and the technology of variability analysis. Crit. Care. 8, R367–R384. 10.1186/cc2948 15566580 PMC1065053

[B23] Silva A. F. Pimenta F. Alves M. A. Oliveira M. S. (2020). Flow dynamics of vitreous humour during saccadic eye movements. J. Mech. Behav. Biomed. Mater. 110, 103860. 10.1016/j.jmbbm.2020.103860 32755799

[B24] Singh D. Firouzbakhsh K. Ahmadian M. T. (2017). Human intraocular thermal field in action with different boundary conditions considering aqueous humor and vitreous humor fluid flow. Int. J. Mech. Mechatron. Eng. 11, 717–725.

[B25] Tabernero J. Artal P. (2014). Lens oscillations in the human eye. Implications for post-saccadic suppression of vision. PLoS One 9, e95764. 10.1371/journal.pone.0095764 24755771 PMC3995773

[B26] Tabernero J. Chirre E. Hervella L. Prieto P. Artal P. (2016). The accommodative ciliary muscle function is preserved in older humans. Sci. Rep. 6, 25551. 10.1038/srep25551/ 27151778 PMC4858807

[B27] Wang K. Pierscionek B. K. (2019). Biomechanics of the human lens and accommodative system: functional relevance to physiological states. Prog. Retin. Eye Res. 71, 114–131. 10.1016/j.preteyeres.2018.11.004 30439450

[B28] Yamagishi S. Yoneya M. Furukawa S. (2020). Relationship of postsaccadic oscillation with the state of the pupil inside the iris and with cognitive processing. J. Neurophysiol. 123, 484–495. 10.1152/jn.00205.2019 31825707 PMC7052648

[B29] Zapata-Díaz J. F. Radhakrishnan H. Charman W. N. López-Gil N. (2019). Accommodation and age-dependent eye model based on *in vivo* measurements. J. Optom. 12, 3–13. 10.1016/j.optom.2018.01.003 29573985 PMC6318498

[B30] Zemax (2024). OpticStudio, version 22.1.1.

